# Seminal Oxidative Stress and Sperm DNA Fragmentation in Men from Couples with Infertility or Unexplained Recurrent Pregnancy Loss

**DOI:** 10.3390/jcm13030833

**Published:** 2024-01-31

**Authors:** Johanne Mejlholm Kold Rasmussen, Maya Isabella Riise Dalgaard, Hiva Alipour, Fereshteh Dardmeh, Ole Bjarne Christiansen

**Affiliations:** 1 Department of Obstetrics and Gynecology, Aalborg University Hospital, 9000 Aalborg, Denmark; johanne.kold@rn.dk (J.M.K.R.); maya.dalgaard@rn.dk (M.I.R.D.); 2 Regenerative Medicine, Department of Health Science and Technology, Aalborg University, 9260 Gistrup, Denmark; feda@hst.aau.dk; 3 Department of Clinical Medicine, Aalborg University, 9260 Gistrup, Denmark

**Keywords:** sperm quality, recurrent pregnancy loss, idiopathic infertility, seminal oxidative stress, sperm DNA Fragmentation

## Abstract

(1) Background: This case–control study examined whether men from couples with unexplained recurrent pregnancy loss (RPL) or infertility exhibited higher seminal oxidative stress (OS) and sperm DNA fragmentation (SDF) compared to fertile controls. (2) Methods: The study included 30 participants from each group: unexplained RPL, unexplained infertility, and proven fertility. Data were collected at Aalborg University Hospital tertiary RPL and fertility treatment clinics (Aalborg, Denmark), excluding couples with mixed conditions for homogeneity. Semen samples were analyzed using computer-aided sperm analysis (CASA) for concentration, motility, and morphology. SDF was assessed via a CASA-based sperm chromatin dispersion test. OS was measured as static oxidation-reduction potential (sORP). (3) Results: The results showed no significant OS differences between groups. The RPL group had significantly lower SDF levels than the control group. A significant positive correlation between SDF and OS was observed in the infertility group. Overall, this study did not find significant differences in OS levels between men from couples with unexplained RPL or infertility and fertile controls, while SDF levels were lower in the RPL group compared to controls. (4) Conclusion: In conclusion, despite the existing literature suggesting that OS and SDF are negative prognostic factors, our findings suggest they may not be reliable diagnostic markers for RPL and infertility.

## 1. Introduction

Involuntary childlessness is a major global problem [1]. Approximately 15% of all couples in their reproductive age experience infertility, defined as “failure to achieve a clinical pregnancy after 12 months or more of regular unprotected sexual intercourse” [1,2], and 1–2% will experience recurrent pregnancy loss (RPL) [3]. Different scientific societies have put forward various definitions of RPL [4]. In this study, RPL was defined as three or more consecutive early pregnancy losses [5]. Approximately 50% of RPLs remain unexplained [3,6]. Increased seminal OS and SDF have been frequently reported in men from couples with infertility or RPL [7,8,9,10,11,12,13].

Oxidative stress (OS) is the state where the level of reactive oxygen species (ROS), resulting from oxygen metabolism, exceeds the environmental antioxidant capacity [14]. Potential sources of ROS in semen include leukocytospermia, immature and dysfunctional spermatozoa, varicocele, genitourinary tract infection, and environmental factors such as smoking and alcohol consumption [14,15]. ROS contribute to many processes at normal physiological levels, including intracellular signaling, leading to maturation, hyperactivation, capacitation, and acrosome reaction in sperm [14]. The occurrence of OS in the microenvironment of the spermatozoa can cause lipid peroxidation, resulting in plasma membrane disturbance, impaired flagellar movement, and reduced energy production, resulting in decreased motility, apoptosis, and increased SDF [14,15,16]. Studies have demonstrated that high SDF and oxidative stress negatively correlate with sperm concentration, motility, vitality, and morphology [7,8,15,16,17].

Despite the reports of increased OS and SDF in men from couples with infertility or RPL, there is still no consensus on whether the assessment of these factors should be considered as routine practice in diagnosing unexplained RPL or infertility [6,18]. Therefore, this comparative study investigated semen parameters, including seminal OS and SDF levels in men from couples with unexplained infertility or unexplained RPL and fertile controls, to investigate their diagnostic value in these disorders.

## 2. Materials and Methods

### 2.1. Ethical Approval

This clinical case–control study was performed at the RPL Centre of Western Denmark and the Fertility Unit, Aalborg University Hospital (Aalborg, Denmark) and the Department of Health Science and Technology, Aalborg University (Aalborg, Denmark) under approval by the Scientific Ethics Committee of the North Jutland Region, Denmark (approval number N-20190023). Participants received written and oral information regarding the study before providing signed consent and filling out a demographic questionnaire. Furthermore, details on the RPL and infertility couples were acquired from the female partner’s hospital journal.

### 2.2. Study Subjects

The present study included men from couples diagnosed with unexplained infertility (*n* = 30), unexplained RPL (*n* = 30), and couples with proven fertility (*n* = 30). The RPL group included men from couples referred to the RPL Centre of Western Denmark, with RPL defined as three or more consecutive confirmed pregnancy losses on or before the 20th week of gestation [5]. The infertility group included men from couples with unexplained primary infertility [19] undergoing treatment at the Fertility Unit of Aalborg University Hospital. The proven fertility group (control group) included men who had fathered at least one child within the last three years without any history of miscarriage, infertility, or use of assisted reproductive technology (ART).

#### General Inclusion and Exclusion Criteria

Only men between 18 and 50 years of age were included in this study. Known genetic abnormality, ejaculatory disorders, cardiovascular disease, metabolic diseases (e.g., diabetes), history of vasectomy, orchitis, orchiectomy, testicular cancer or chemotherapy, and malignant diseases within the last five years were considered as exclusion criteria. Use of antibiotics, antifungal agents, antidepressants and other psychopharmacological treatments, cimetidine, cyclosporine, colchicine, allopurinol, or systemic glucocorticoids, or the presence of acute infection within the last three months led to exclusion as well. Men from couples with infertility or RPL were excluded if the women had an irregular menstrual cycle (<23 or >35 days) or proven tubal or uterine abnormality.

Men in the infertility group were excluded if they had a preliminary semen quality below the WHO reference values [20] detected in the Fertility Unit at Aalborg University Hospital or if the couple had a history of miscarriage. Men in the RPL group were excluded if the couple was undergoing fertility treatment at the time of the study or had not achieved spontaneous conception during the last 12 months. Men in the RPL group were also excluded if the female partners were lupus anticoagulant positive or had high levels of the antiphospholipid antibodies (IgG or IgM anticardiolipin or β2-glycoprotein I antibodies (>30 IU/L)) in the blood. Other medical conditions of the female partner, namely diabetes or hypothyroidism, were not considered as exclusion criteria provided that they were under control.

### 2.3. Semen Analysis

Semen samples were collected by masturbation, weighted to determine volume [20], and allowed to liquefy at room temperature. To not interfere with the treatment, only 200 μL of the semen sample from the infertility group was collected for this study, while the remainder was used for ART. Samples were analyzed to determine SDF, morphology, concentration, motility, and kinematic parameters according to criteria defined in the WHO 2021 guidelines [19], using a Nikon Eclipse E200 microscope (Nikon, Tokyo, Japan) equipped with a Basler Scout A780 camera (Basler, Ahrensburg, Germany) and the Sperm Class Analyzer (SCA^®^; Version 6.5, Microptic S.L., Barcelona, Spain) computer-aided semen analysis (CASA) software.

Semen samples were assessed for concentration, motility, and morphology using the Sperm Class Analyzer (SCA) computer-aided sperm analysis (CASA) system. For each sample, 2.5 μL of liquified semen was transferred into a Leja slide chamber (20 microns depth, Leja, Nieuw-Vennep, The Netherlands). A minimum of 5 different fields was assessed using a 10× positive phase objective and a green filter for concentration, motility, and kinematic parameters, including curvilinear velocity (VCL), average path velocity (VAP), straight-line velocity (VSL), the amplitude of lateral head displacement (ALH), beat cross frequency (BCF), straightness (STR), linearity (LIN), and wobble (WOB), as previously described by Alipour et al. [21].

Progressive motility was defined as the percentage of spermatozoa with STR > 80%. Rapid progressive motility was defined as the percentage of sperm cells with a forward progression of >25 µm/s, as described by WHO 2021 criteria [19]. Mucus penetration ability was defined as the number of sperm cells with VAP > 25 μm s^−1^, STR > 80%, and ALH > 2.5 μm in the liquified seminal plasma [22].

To assess morphology, 10 μL of liquified semen from each sample was used to prepare a seminal smear. The smear was air-dried before being fixed (10 min) and stained (10 min) using the SpermBlue morphology staining kit (Microptic, Barcelona, Spain). The percentage of spermatozoa with normal morphology was then evaluated using a 60× objective (brightfield), and sperm with a normal morphology ≥ 4% were considered normal, according to WHO 2010 reference values [20].

According to WHO 2010 reference values, concentration ≥ 15 million/mL, sperm count ≥ 39 million/sample, total motility ≥ 40%, progressive motility ≥ 32%, and morphology ≥ 4% were considered normal [20].

Seminal OS was measured as static oxidation-reduction potential (sORP) by assessing 30 μL of each sample using the Male Infertility Oxidative System (MiOXSYS) (Caerus Biotech, Vienna, Switzerland). sORP was normalized based on sperm concentration, and normalized sORP values above 1.38 mV/10^6^ sperm mL were considered pathologic [23].

SDF was assessed based on the Sperm Chromatin Dispersion (SCD) test [24] using the GoldCyto Sperm DNA kit (Microptic, Barcelona, Spain). A minimum of 200 spermatozoa were observed using a 20× objective (brightfield), and halo sizes were evaluated. SDF values above 15% were considered pathologic.

### 2.4. Statistical Analysis

Data were checked for normal distribution using the Shapiro–Wilk test. Normally distributed data are presented as mean ± standard deviation (SD), and non-normally distributed data as median (first quartile–third quartile). A between-group comparison was performed using ANOVA with Bonferroni correction. Analysis of covariance (ANCOVA) was performed to adjust for potential confounders, including abstinence and analysis time (time from ejaculation until semen analysis), in seminal parameters that may have been affected by these factors (see Supplementary Table S1). Fisher’s exact test was used to compare binary variables. Correlations between the semen parameters were tested using Spearman’s rank correlation coefficient. IBM Statistical Package for Social Sciences (SPSS) (Ver. 26, IBM Corporation, Armonk, NY, USA) was used to perform the statistical analyses, and *p* < 0.05 was considered significant. Due to the low sample size, borderline differences at a 10% significance level were also reported.

## 3. Results

All participants completed the study, and no safety issues were reported. Characteristics of the three groups of participants are shown in Table 1. The RPL and infertility groups comprised a significantly higher (*p* < 0.05) percentage of smokers than controls. Both men and women in the RPL group were significantly (*p* < 0.01) older than the control group. No significant differences in BMI, amount of exercise, amount of alcohol, or percentage of participants taking dietary supplementation were observed among the groups. Types of dietary supplementation registered in the groups were multivitamin pills, vitamins B, C, D, and E, calcium, magnesium, zinc, selenium, calcium, coenzyme Q10, creatinine, taurine, curcumin, ginger, fish oil, and probiotics.

Couples in the control group had conceived after 2.4 ± 2.9 (mean ± SD) months, while the infertility group had tried to conceive for 30.7 ± 22.3 months, and the RPL group had tried to achieve birth for 30.3 ± 23.8 months. In the control group, 60% of the participants had one child, and the remaining had more children. Of the couples in the RPL group, 66.7% had experienced primary RPL (no children), while 33.3% had had one or more children before RPL. On average, couples in the RPL group had experienced 3.8 ± 1.1 consecutive pregnancy losses; 56.7% had experienced three while the remaining had experienced 4–6 consecutive pregnancy losses.

### 3.1. Semen Parameters

The semen analysis results adjusted for confounders are shown in Table 2. The mean SDF% in all three groups was within the normal range (>20%) [25], but significantly lower in the RPL group compared to the control group. No significant differences were observed when comparing SDF in the infertility group with the RPL and control groups. There was no significant difference in sORP level between the groups.

Volume and concentration showed no significant differences between the three groups. The percentage of sperm with normal morphology was higher in the RPL than in the infertility (*p* < 0.05) and control (*p* < 0.10) groups. The infertility group had lower (*p* < 0.10) total motility compared to both control and RPL groups and a lower (*p* < 0.10) total sperm count and progressive motility than the controls.

In the infertility group, 73.3% of the participants had lower total motility, while 86.7% had lower progressive motility than the WHO reference values [20]; these values were 40.0% and 50.0% in the RPL group and 23.3% and 46.7% in the control group, respectively. The three groups showed similar percentages of participants with concentration, sperm count, and volume above the WHO reference values [20]. More detailed sperm kinematic parameters assessed by CASA are shown in Supplementary Table S2 in the supplementary files.

#### Correlation between Semen Parameters

Correlation coefficients (r) between semen parameters in the different groups are shown in Table 3. A significantly positive correlation between SDF and normalized sORP was observed in infertility (r = 0.51, *p* = 0.004) and RPL (r = 0.37, *p* = 0.05) groups. Morphology was negatively correlated to normalized sORP in infertility and control groups (r = −0.60, *p* < 0.001 and r = −0.44, *p* = 0.015, respectively). Motility was negatively correlated with SDF in all groups and with normalized sORP in the infertility group.

## 4. Discussion

The present study found no difference in OS levels among the control, RPL, and infertility groups but revealed a lower SDF in the RPL group than in the control and unexplained infertility groups [7,8,10,13,26].

A recent review on the role of seminal oxidative stress in recurrent pregnancy loss by Davies et al. [4] reported that the evidence regarding the association between ROS and SDF in RPL patients is not universally supported and remains controversial. Indeed, our findings were in contrast with previous studies reporting high levels of SDF and oxidative stress among men from infertile and RPL couples compared to controls [7,8,10,13,26]. This study also identified significant negative correlations between SDF and motility in both the RPL and infertility groups and a significant negative correlation between OS and motility in the infertility group, supporting results from previous studies [4,15].

The lower (*p* < 0.10) total motility in the infertility group compared to both control and RPL groups was expected, as sperm motility is considered one of the key predictors of male infertility [27]. The present study focused on RPL couples without concomitant infertility problems and a control group with proven fertility and no miscarriages, diverging it from the previous studies assessed by Davies et al. [4]. Thus, the higher prevalence of SDF in RPL than in controls demonstrated in the previous studies could be related to a concomitant motility-related infertility problem or a heterogenous control group rather than the RPL problem per se. The well-defined groups could potentially explain why the present study did not demonstrate increased SDF and OS in the RPL group, supporting the idea that heterogeneity amongst the study designs may be accountable for the conflicting findings [4].

A meta-analysis by Tan et al. [13] including 13 studies of RPL couples found a significantly higher level of SDF among RPL couples compared to controls and a significant heterogeneity among the studies included. The meta-analysis differed from the present study by including a predominance of studies with RPL defined as two or more miscarriages, and some included controls and infertile couples with a history of miscarriage. Some of the previous studies excluding RPL couples with infertility did not find a higher frequency of SDF in the RPL group compared to controls [28,29], whereas other studies did [30,31]. Thus, the possible potential of SDF as a reliable diagnostic factor for RPL remains controversial, emphasizing the need for further, more extensive studies of homogeneous patients and control groups to elucidate the diagnostic value of high SDF for RPL. A study by Zhang et al. indicated that SDF may be considered a prognostic factor regarding the reproductive outcome of RPL couples rather than a possible diagnostic tool for RPL [28]. Further investigation on the clinical applicability of SDF and OS in prognosticating RPL cases is required before a solid conclusion can be drawn [28].

ROS, OS, and SDF are interrelated [14,15]. The present study also found a strong correlation between OS and SDF in the infertility group, while a tendency towards a positive correlation was observed in the RPL group, consistent with the literature [9,12,13,15]. A study by Ribas-Maynou and Benet suggested that different forms of SDF, including single-strand breaks, extensive double-strand breaks, and localized double-strand breaks, are connected to different clinical conditions and should be considered separately [32]. Localized double-strand breaks were reported to be associated with an increased risk of miscarriages [32]. Ribas-Maynou et al. found that 85% of men in RPL couples had a low level of single-strand breaks and a high level of double-strand breaks compared with only 33% of fertile male controls [33]. In contrast, single-strand breaks were reported to be related to OS, a longer time to conception, and infertility [32]. The SCD test used in this study for SDF detection reveals single-strand breaks and may reveal extensive double-strand breaks but does not make it possible to differentiate between the different types of SDF [32]. Thus, it is possible that the type of SDF detected in controls and RPL may be a mixture of different SDF types and thereby only weakly correlated to the presence of OS in the seminal fluid. In contrast, the SDF detected in the infertility group could be primarily single-strand breaks and, therefore, more strongly correlated to OS.

The RPL and Infertility groups included more smokers than the control group (35.0%, 36.4%, and 3.4%, respectively). While smoking is a known risk factor for seminal ROS [14], a higher level of normalized sORP in the RPL and infertility groups was not observed compared to the control group. A meta-analysis [34] reported passive smoking to be related to an increased risk of miscarriages, which aligns with our findings regarding the higher number of smokers in infertility and RPL groups. However, our study does not support the theory that the pathway between male smoking and unexplained infertility involves increased seminal ROS.

In this study, the percentage of men taking dietary supplementation and herbal medicine was 20% less in the RPL group (23%) than in the infertility (43%) and control groups (44%); this difference was not significant, and the participants were not instructed to follow any special diet prior to the semen analysis that could have been expected to affect the semen quality analysis including the OS or SDF analysis. While SDF in all three groups was within the normal range, the lower SDF and OS (although not significant) in the RPL group might be attributable to a potentially healthier lifestyle and diet recommended during their treatment course, which was not considered in this study.

The lower OS in the RPL group can also explain the higher percentage of sperm with normal morphology in this group, as OS has been reported to lead to axonemal impairment and increased morphological defects in the midpiece of sperm [35].

Furthermore, the mean age of both the female partner and the men in the RPL group was significantly higher than in the control group in the present study. Advanced paternal age has been positively correlated to SDF [36]. Despite the significantly higher age in the RPL group, the SDF levels were significantly lower in the RPL group compared to the control group, which may be due to the mean age difference (around four years) being too small to influence the SDF levels.

In the present study, semen parameters were adjusted for the difference in analysis and abstinence times when considered relevant using ANCOVA. Ayad et al. [37] concluded that an increase in abstinence time increases concentration and volume, while a decrease in abstinence time improves motility parameters. Regarding analysis time, a study has demonstrated that motility decreases 5–10% per hour after ejaculation [38]. Thus, the literature supports the necessity of taking abstinence and analysis time into account when considering semen parameters.

The present study aimed to examine semen from men in couples with unexplained RPL or unexplained infertility, the latter diagnosis based on a preliminary manual routine sperm analysis. However, 73.3% of the men in the infertility group had a percentage of total motile sperm below the reference values set by the WHO guidelines [20] when samples were analyzed using the CASA system. Manual analysis results greatly depend on the technician’s level of training and momentary performance, while CASA is more accurate, objective, and reproducible [39,40]. Thus, many couples diagnosed with unexplained infertility in the present study may have an undiscovered male factor as part of their infertility problem.

## 5. Conclusions

To the authors’ knowledge, this is the first study comparing semen quality, including seminal OS and SDF, between ensured very pure and homogeneous groups of men from couples with unexplained RPL, unexplained infertility, and proven fertility. Seminal OS did not differ significantly between the study groups. SDF was not different between infertility and other groups, while it was surprisingly significantly lower in the RPL group compared to the control group, indicating that SDF might not be a reliable diagnostic factor for RPL and infertility. Further studies with long-term follow-up are needed to draw a more solid conclusion on the importance of SDF and seminal OS in diagnosing, prognosis, and treating unexplained RPL and infertility patients.

## Figures and Tables

**Table 1:** Characteristics of men in the recurrent pregnancy loss (RPL), idiopathic infertility (infertile), and proven fertility (control) groups.

Characteristic	RPL	Infertile	Controls	*p*-Value (RPL-Infertile)	*p*-Value (RPL-Controls)	*p*-Value (Infertile-Controls)
Age (years)	34.7 (30.1–40.0)	30.7 (29.8–36.3)	31.3 (29.2–33.1)	0.35	0.008 *	0.41
Body mass index (kg/m^2^)	25.6 (23.7–29.8)	25.6 (23.1–27.8)	26.0 (23.6–28.3)	1.00	1.00	1.00
Exercise (hours/week)	2.5 (0.0–4.5)	2.5 (1.0–3.5)	2.5 (1.0–5.0)	1.00	1.00	1.00
Alcohol (units/week)	3.5 (1.0–10.0)	2.5 (1.0–6.5)	1.75 (1.0–5.5)	0.87	0.18	1.00
Smokers (%)	26.7%	31.0%	3.3%	0.78	0.026 *	0.006 *
Dietary supplementation (%)	23.3%	44.8%	43.3%	0.10	0.17	1.00
Age of female partner (years)	32.2 (28.5–36.1)	30.1 (29.2–31.4)	29.7 (27.2–31.4)	0.17	0.004 *	0.49

The percentage of smokers, median (25–75 percentiles) age, body mass index, exercise, alcohol consumption, and age of the female partners are shown for each group. Asterisks mark significantly different pairwise (between-group) comparisons (*p* < 0.05). Data show no significant difference in BMI, exercise, alcohol, or dietary supplementation between groups, significantly higher male and female age in RPL couples compared to controls, and a significantly higher percentage of male smokers in infertile couples and controls compared to RPL couples.

**Table 2:** Seminal parameters in the recurrent pregnancy loss (RPL), idiopathic infertility (infertile), and proven fertility (control) groups. Parametric data are shown as means ± standard deviation, and nonparametric data as medians (25–75 percentiles). Comparisons adjusted for abstinence and analysis time are presented in { }. Comparisons adjusted for abstinence time are presented in [ ]. Asterisks mark significantly different pairwise (between-group) comparisons (*p* < 0.05).

Parameter	RPL	Infertile	Controls	*p*-Value (RPL-Infertile)	*p*-Value (RPL-Controls)	*p*-Value (Infertile-Controls)
SDF (%)	13.58 (11.71–19.37)	16.33 (12.78–22.25)	16.65 (14.03–23.67)	{0.44}	{0.020} *	{0.37}
Normalized sORP (mV/10^6^ sperm mL)	1.04 (0.64–2.03)	1.54 (0.78–2.36)	1.68 (0.70–2.58)	{0.073}	{0.16}	{0.12}
sORP (mV)	41.98 ± 17.23	44.62 ± 19.72	56.22 ± 19.64	{0.62}	{0.013} *	{0.071}
Volume (mL)	3.8 ± 1.5	3.7 ± 1.4	4.3 ± 1.5	[0.47]	[0.36]	[0.19]
Concentration (×10^6^/mL)	31.42 (23.03–51.10)	28.17 (17.56–42.17)	36.76 (21.51–66.00)	[0.48]	[0.60]	[0.18]
Total sperm count (×10^6^)	134.25 (78.76–189.74)	106.34 (53.14–165.20)	164.14 (91.98–250.85)	[0.28]	[0.59]	[0.054]
Morphology (%)	4.4 (2.9–6.9)	2.9 (1.9–4.9)	2.9 (1.9–4.9)	[0.037] *	[0.083]	[0.54]
Total motility (%)	51.97 (34.32–69.42)	25.88 (18.09–40.19)	54.38 (40.45–69.19)	{0.099}	{0.66}	{0.056}
Progressive motility (%)	30.17 (15.52–48.88)	11.18 (5.12–20.56)	32.54 (24.44–43.95)	{0.12}	{0.83}	{0.073}
Rapid progressive motility (%)	6.39 (2.04–10.89)	1.86 (0.28–3.88)	4.76 (1.99–13.46)	{0.47}	{0.97}	{0.42}
Mucus penetration ability (×10^6^)	7.29 (1.42–15.19)	1.14 (0.16–4.71)	5.54 (1.83–20.25)	{0.99}	{0.41}	{0.28}

When adjusted for abstinence time or abstinence and analysis time, data show significantly lower SDF and sORP in semen from men from RPL couples compared to controls, significantly better morphology in semen from men from RPL couples compared to infertile couples, and no significant differences in normalized sORP, volume, concentration, total sperm count, or motility parameters between groups.

**Table 3:** Correlation coefficients (r) and *p*-values between sperm DNA fragmentation (SDF) and normalized static oxidation-reduction potential (sORP) in all participants, the recurrent pregnancy loss (RPL), idiopathic infertility (infertile), and proven fertility (control) groups.

Correlation	All		RPL		Infertile		Controls	
	r	*p*-Value	r	*p*-Value	r	*p*-Value	r	*p*-Value
SDF—Normalized sORP	0.36	<0.001 *	0.37	0.050	0.51	0.004 *	0.13	0.50
SDF—Volume	−0.02	0.87	−0.06	0.77	0.22	0.25	0.24	0.21
Normalized sORP—Volume	0.17	0.11	0.04	0.85	0.23	0.22	0.18	0.35
SDF—Concentration	−0.25	0.017 *	−0.23	0.22	−0.45	0.012 *	−0.08	0.70
Normalized sORP—Concentration	−0.86	<0.001 *	−0.82	<0.001 *	−0.86	<0.001 *	−0.92	<0.001 *
SDF—Morphology	−0.27	0.010 *	−0.22	0.24	−0.29	0.12	−0.28	0.13
Normalized sORP—Morphology	−0.41	<0.001 *	−0.07	0.73	−0.60	<0.001 *	−0.44	0.015 *
SDF—Total motility	−0.44	<0.001 *	−0.42	0.021 *	−0.53	0.003 *	−0.47	0.009 *
Normalized sORP—Total motility	−0.26	0.016 *	0.05	0.80	−0.68	<0.001 *	−0.19	0.33

Asterisks mark significantly different pairwise (between-group) comparisons (*p* < 0.05). Data show significant correlations between SDF and normalized sORP, SDF and concentration, normalized sORP and concentration, SDF and morphology, normalized sORP and morphology, SDF and motility, as well as normalized sORP and motility in some groups, but no significant correlation between SDF and volume or normalized sORP and volume.

## Data Availability

The data supporting this study’s findings are available from the corresponding authors, [O.B.C.], upon reasonable request.

## Supplementary Materials

The following supporting information can be downloaded at: https://www.mdpi.com/article/10.3390/jcm13030833/s1, Table S1: Abstinence time and analysis time (time from ejaculation until semen analysis) in the recurrent pregnancy loss (RPL), idiopathic infertility (infertile), and the proven fertility (control) groups; Table S2: Supplementary semen parameters in the recurrent pregnancy loss (RPL), idiopathic infertility (infertile), and proven fertility (control) groups.
