# Supplementary material for: Seminal Oxidative Stress and Sperm DNA Fragmentation in Men from Couples with Infertility or Unexplained Recurrent Pregnancy Loss
Source: J Clin Med. 2024 Jan 31;13(3):833. doi: 10.3390/jcm13030833 (PMC10856715; doi:10.3390/jcm13030833)
Supplement: Supplementary file 1 [file jcm-13-00833-s001.zip › jcm-2803081-supplementary.pdf]

Supplementary file

Table S1: Abstinence time and analysis time (time from ejaculation until semen analysis) in the recurrent pregnancy loss (RPL), idiopathic infertility (infertile) and the proven fertility (control) groups.

Parameter	RPL	Infertile	Controls	p-value (RPL-infertile)	p-value (RPL-controls)	p-value (Infertile-controls)
Abstinence time	48 (39-61)	48 (44-68)	60 (48-80)	0.36	0.026 *	0.13
Analysis time for sORP	56 (51-81)	72 (55-85)	50 (45-61)	0.34	0.052	0.006 *
Analysis time for count and motility	58 (52-81)	110 (95-128)	56 (45-75)	< 0.001 *	0.18	< 0.001 *
Analysis time for morphology	67 (52-83)	120 (89-147)	71 (50-97)	< 0.001 *	0.89	< 0.001 *
Analysis time for SDF	95 (60-112)	140 (115-154)	88 (66-115)	< 0.001 *	0.99	< 0.001 *

Parametric data are shown as means ± standard deviation and non-parametric data as medians (25-75 percentiles). Data have been presented in minutes. Asterisks mark significantly different pairwise (between-group) comparisons (p<0.05). Data shows that abstinence time was significantly shorter in the RPL group compered to controls, analysis time for sORP was significantly longer in the infertile group compared to controls, and that analysis time for count, motility, morphology and SDF was significantly longer in the infertile group compared to both the RPL group and controls.

Table S2: Supplementary semen parameters in the recurrent pregnancy loss (RPL), idiopathic infertility (infertile) and proven fertility (control) groups.

**Parameter**	**RPL**	**Infertile**	**Controls**	**p-value (RPL-infertile)**	**p-value (RPL-controls)**	**p-value (infertile-controls)**
VCL (μm/s)	39.73 (29.93-45.19)	29.23 (22.72-35.83)	35.41 (30.73-44.32)	< 0.001 *	0.61	0.001 *
VAP (μm/s)	22.84 ± 4.20	18.14 ± 3.88	22.45 ± 4.32	< 0.001 *	0.73	< 0.001 *
VSL (μm/s)	14.21 ± 3.07	10.89 ± 3.56	14.27 ± 3.40	< 0.001 *	0.94	< 0.001 *
STR (%)	58.69 ± 5.39	55.31 ± 9.32	60.12 ± 6.30	0.090	0.35	0.023
LIN (%)	36.99 ± 4.87	35.97 ± 6.75	38.10 ± 6.51	0.50	0.46	0.22
WOB (%)	61.26 ± 4.98	63.71 ± 5.69	61.25 ± 5.99	0.082	0.99	0.11
ALH (μm)	1.96 (1.59-2.29)	1.60 (1.38-1.83)	1.79 (1.62-2.29)	< 0.001 *	0.76	0.001 *
BCF (Hz)	5.71 ± 0.87	4.87 ± 1.24	5.67 ± 0.99	0.003 *	0.85	0.007 *
Head area (μm^2)	7.52 ± 1.07	7.61 ± 0.93	7.59 ± 0.88	0.74	0.82	0.91
Round cells ($x10^6$/ml)	0.26 (0.80-46.0)	0.25 (0.90-0.51)	0.33 (0.11-0.71)	0.88	0.43	0.65

Parametric data are shown as means ± standard deviation, and non-parametric data as medians (25-75 percentiles). None of the parameters has been adjusted for confounding factors. Asterisks mark significant correlations (p<0.05). Data shows that there was no difference between the groups regarding STR, LIN, WOB, spermatozoa head area or the number of round cells in the semen, but that VCL, VAP, VSL, ALH and BCF were significantly lower in the infertile groups compared to both the RPL group and controls, respectively.
